# POSITIVE AND NEGATIVE RELATIONSHIPS, OUTLOOK, AND DEPRESSION PREDICT LONELINESS IN OLDER ADULTS DURING COVID-19

**DOI:** 10.1093/geroni/igac059.1900

**Published:** 2022-12-20

**Authors:** Benjamin Hougaard, Vennisia Mo, Anahita Eskandari, Rowena Gomez

**Affiliations:** Palo Alto University, Mountain View, California, United States; Palo Alto University, Palo Alto, California, United States; Palo Alto University, Palo Alto, California, United States; Palo Alto University, Palo Alto, California, United States

## Abstract

The COVID-19 pandemic has changed the way many older adults live and function in the United States. Because of the isolation from stay-at-home mandates and higher risk of death from infection, many older adults are experiencing depressive symptoms, anxiety, and increased loneliness (National Council on Aging [NCOA], 2021; Center for Disease Control [CDC], 2021). One factor might be whether one has a spouse, children, or friends and if those relationships are positive or negative (Vanderhorst & McLaren, 2005). Besides the quality and nature of the relationship, another factor might be the amount of virtual contact while in isolation during COVID-19 (Regis College Online, 2020.In this study, we examined the relationships between outlook (optimism, pessimism, hopelessness), depression, loneliness, and social relationships in older adults during the COVID-19 pandemic in a sample of 808 adults 60 years of age or older. The data was gathered from self-reported questionnaires from the psychosocial and lifestyle questionnaire and health questionnaire from the Health and Retirement Study (HRS) 2020 version (HRS, 2021).We found that loneliness scores were significantly predicted by self-reported measures of positive and negative relationships, along with optimism, pessimism, hopelessness, depressive symptom scores, and COVID-19 life changes (Adjusted R2 = .447, F(12, 795) = 55.26, p < .001). COVID-19 has changed the way many older adults live and function. Finding ways to increase the level of communication older adults are able to have with their close relationships is crucial to combating this epidemic of loneliness.

